# *N*′-(4-Bromo­benzyl­idene)thio­phene-2-carbohydrazide

**DOI:** 10.1107/S1600536810010603

**Published:** 2010-03-27

**Authors:** Jin-He Jiang

**Affiliations:** a Microscale Science Institute, Department of Chemistry and Chemical Engineering, Weifang University, Weifang 261061, People’s Republic of China

## Abstract

In the title compound, C_12_H_9_BrN_2_OS, the dihedral angle between the aromatic rings is 10.0 (2)°. In the crystal structure, inversion dimers linked by pairs of N—H⋯O hydrogen bonds occur, generating *R*_2_^2^(8) loops. Weak aromatic π–π stacking [centroid–centroid separations = 3.825 (3) and 3.866 (3) Å] also occurs.

## Related literature

For background to Schiff bases, see: Cimerman *et al.* (1997 ▶). For a related structure, see: Girgis (2006 ▶).
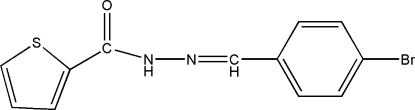


## Experimental

### 

#### Crystal data

C_12_H_9_BrN_2_OS
*M* *_r_* = 309.18
Monoclinic, 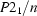

*a* = 6.0700 (12) Å
*b* = 16.983 (3) Å
*c* = 11.643 (2) Å
β = 94.85 (3)°
*V* = 1195.9 (4) Å^3^
*Z* = 4
Mo *K*α radiation
μ = 3.60 mm^−1^
*T* = 293 K
0.23 × 0.20 × 0.18 mm

#### Data collection

Bruker SMART CCD diffractometer
8238 measured reflections
2079 independent reflections
1614 reflections with *I* > 2σ(*I*)
*R*_int_ = 0.086

#### Refinement

*R*[*F* ^2^ > 2σ(*F* ^2^)] = 0.054
*wR*(*F* ^2^) = 0.163
*S* = 1.03
2079 reflections
154 parameters
H-atom parameters constrained
Δρ_max_ = 0.75 e Å^−3^
Δρ_min_ = −0.84 e Å^−3^

### 

Data collection: *SMART* (Bruker, 1997 ▶); cell refinement: *SAINT* (Bruker, 1997 ▶); data reduction: *SAINT*; program(s) used to solve structure: *SHELXS97* (Sheldrick, 2008 ▶); program(s) used to refine structure: *SHELXL97* (Sheldrick, 2008 ▶); molecular graphics: *SHELXTL* (Sheldrick, 2008 ▶); software used to prepare material for publication: *SHELXTL*.

## Supplementary Material

Crystal structure: contains datablocks global, I. DOI: 10.1107/S1600536810010603/hb5368sup1.cif

Structure factors: contains datablocks I. DOI: 10.1107/S1600536810010603/hb5368Isup2.hkl

Additional supplementary materials:  crystallographic information; 3D view; checkCIF report

## Figures and Tables

**Table 1:** Hydrogen-bond geometry (Å, °)

*D*—H⋯*A*	*D*—H	H⋯*A*	*D*⋯*A*	*D*—H⋯*A*
N1—H1*A*⋯O1^i^	0.86	2.00	2.828 (5)	161

Symmetry code: (i) 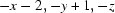.

**Fig. 1.**
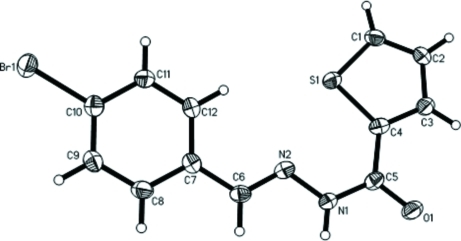
The molecular structure of (I) showing 30% probability displacement ellipsoids.

## supplementary crystallographic information

### Comment

Schiff bases have received considerable attention in the literature. They are attractive from several points of view, such as the possibility of analytical application (Cimerman *et al.*, 1997). As part of our search for new Schiff base compounds we synthesized the title compound (I), and describe its structure here.

The molcular structure of (I) is shown in Fig. 1. The C6—N2 bond length of 1.273 (3)Å is comparable with C—N double bond [1.281 (2) Å] reported (Girgis, 2006). In the crystal structure, molecules are linked by intermolecular N—H···O hydrogen bonds.

### Experimental

A mixture of thiophene-2-carbohydrazide (0.05 mol), and 4-bromobenzaldehyde (0.05 mol) was stirred in refluxing ethanol (10 mL) for 4 h to afford the title compound (0.080 mol, yield 80%). Colourless blocks of (I) were obtained by recrystallization from ethanol at room temperature.

### Refinement

H atoms were fixed geometrically and allowed to ride on their attached atoms, with C—H distances = 0.93-0.97 Å; N—H = 0.86Å and with *U*_iso_(H) = 1.2*U*_eq_(C,*N*) or 1.5*U*_eq_(C_methyl_).

### Crystal data

**Table:**

C_12_H_9_BrN_2_OS	*F*(000) = 616
*M**_r_* = 309.18	*D*_x_ = 1.717 Mg m^−^^3^
Monoclinic, *P*2_1_/*n*	Mo *K*α radiation, λ = 0.71073 Å
Hall symbol: -P 2yn	Cell parameters from 1614 reflections
*a* = 6.0700 (12) Å	θ = 3.5–25.3°
*b* = 16.983 (3) Å	µ = 3.60 mm^−^^1^
*c* = 11.643 (2) Å	*T* = 293 K
β = 94.85 (3)°	Block, colorless
*V* = 1195.9 (4) Å^3^	0.23 × 0.20 × 0.18 mm
*Z* = 4	

### Data collection

**Table:**

Bruker SMART CCD diffractometer	1614 reflections with *I* > 2σ(*I*)
Radiation source: fine-focus sealed tube	*R*_int_ = 0.086
graphite	θ_max_ = 25.3°, θ_min_ = 3.5°
ω scans	*h* = −6→7
8238 measured reflections	*k* = −19→19
2079 independent reflections	*l* = −13→13

### Refinement

**Table:**

Refinement on *F*^2^	Primary atom site location: structure-invariant direct methods
Least-squares matrix: full	Secondary atom site location: difference Fourier map
*R*[*F*^2^ > 2σ(*F*^2^)] = 0.054	Hydrogen site location: inferred from neighbouring sites
*wR*(*F*^2^) = 0.163	H-atom parameters constrained
*S* = 1.03	*w* = 1/[σ^2^(*F*_o_^2^) + (0.1*P*)^2^] where *P* = (*F*_o_^2^ + 2*F*_c_^2^)/3
2079 reflections	(Δ/σ)_max_ < 0.001
154 parameters	Δρ_max_ = 0.75 e Å^−^^3^
0 restraints	Δρ_min_ = −0.84 e Å^−^^3^

### Special details

**Table:**

Geometry. All esds (except the esd in the dihedral angle between two l.s. planes) are estimated using the full covariance matrix. The cell esds are taken into account individually in the estimation of esds in distances, angles and torsion angles; correlations between esds in cell parameters are only used when they are defined by crystal symmetry. An approximate (isotropic) treatment of cell esds is used for estimating esds involving l.s. planes.
Refinement. Refinement of *F*^2^ against ALL reflections. The weighted *R*-factor wR and goodness of fit *S* are based on *F*^2^, conventional *R*-factors *R* are based on *F*, with *F* set to zero for negative *F*^2^. The threshold expression of *F*^2^ > σ(*F*^2^) is used only for calculating *R*-factors(gt) etc. and is not relevant to the choice of reflections for refinement. *R*-factors based on *F*^2^ are statistically about twice as large as those based on *F*, and *R*- factors based on ALL data will be even larger.

### Fractional atomic coordinates and isotropic or equivalent isotropic displacement parameters (Å^2^)

**Table:**

	*x*	*y*	*z*	*U*_iso_*/*U*_eq_	
Br1	0.15443 (9)	0.87907 (3)	0.25958 (4)	0.0509 (3)	
S1	−0.8655 (2)	0.61910 (7)	0.37457 (11)	0.0437 (4)	
C6	−0.5332 (9)	0.6311 (2)	0.0959 (4)	0.0382 (11)	
H6A	−0.5211	0.6061	0.0256	0.046*	
N2	−0.6908 (7)	0.61169 (19)	0.1589 (3)	0.0390 (9)	
C10	−0.0606 (8)	0.8041 (3)	0.2068 (4)	0.0396 (11)	
N1	−0.8383 (6)	0.5559 (2)	0.1135 (3)	0.0390 (9)	
H1A	−0.8193	0.5353	0.0475	0.047*	
O1	−1.1473 (6)	0.4869 (2)	0.1217 (3)	0.0526 (9)	
C5	−1.0117 (8)	0.5329 (2)	0.1702 (4)	0.0401 (11)	
C1	−1.0383 (9)	0.6136 (3)	0.4824 (5)	0.0466 (13)	
H1B	−1.0099	0.6383	0.5534	0.056*	
C3	−1.2229 (8)	0.5388 (3)	0.3431 (4)	0.0431 (11)	
H3A	−1.3366	0.5075	0.3099	0.052*	
C11	−0.2387 (9)	0.7887 (3)	0.2738 (4)	0.0456 (12)	
H11A	−0.2529	0.8163	0.3418	0.055*	
C7	−0.3756 (7)	0.6916 (2)	0.1350 (4)	0.0363 (10)	
C12	−0.3902 (8)	0.7326 (3)	0.2374 (4)	0.0449 (12)	
H12A	−0.5061	0.7217	0.2822	0.054*	
C4	−1.0404 (8)	0.5594 (2)	0.2876 (4)	0.0381 (10)	
C9	−0.0451 (8)	0.7655 (2)	0.1054 (4)	0.0430 (11)	
H9A	0.0705	0.7767	0.0604	0.052*	
C8	−0.2019 (9)	0.7093 (3)	0.0693 (4)	0.0445 (12)	
H8A	−0.1903	0.6832	−0.0001	0.053*	
C2	−1.2204 (8)	0.5698 (2)	0.4544 (4)	0.0433 (11)	
H2B	−1.3312	0.5612	0.5034	0.052*	

### Atomic displacement parameters (Å^2^)

**Table:**

	*U*^11^	*U*^22^	*U*^33^	*U*^12^	*U*^13^	*U*^23^
Br1	0.0496 (5)	0.0514 (4)	0.0511 (5)	−0.01021 (19)	0.0003 (3)	0.00256 (19)
S1	0.0465 (8)	0.0495 (8)	0.0352 (8)	−0.0095 (5)	0.0039 (6)	−0.0090 (5)
C6	0.049 (3)	0.036 (2)	0.029 (3)	0.002 (2)	0.002 (2)	0.0009 (17)
N2	0.046 (3)	0.0360 (19)	0.034 (2)	−0.0016 (16)	−0.0017 (18)	−0.0028 (15)
C10	0.042 (3)	0.038 (2)	0.039 (3)	0.0005 (19)	0.002 (2)	0.0079 (18)
N1	0.041 (2)	0.040 (2)	0.036 (2)	−0.0064 (17)	0.0026 (17)	−0.0082 (15)
O1	0.060 (3)	0.055 (2)	0.042 (2)	−0.0194 (17)	0.0048 (17)	−0.0114 (15)
C5	0.047 (3)	0.034 (2)	0.039 (3)	−0.006 (2)	−0.001 (2)	−0.0020 (18)
C1	0.050 (3)	0.055 (3)	0.035 (3)	0.003 (2)	0.007 (2)	−0.009 (2)
C3	0.044 (3)	0.046 (3)	0.039 (3)	−0.010 (2)	0.006 (2)	−0.005 (2)
C11	0.051 (3)	0.049 (3)	0.037 (3)	−0.008 (2)	0.009 (2)	−0.008 (2)
C7	0.036 (3)	0.038 (2)	0.035 (3)	0.0043 (19)	0.0031 (19)	0.0085 (18)
C12	0.037 (3)	0.051 (3)	0.048 (3)	−0.007 (2)	0.012 (2)	−0.004 (2)
C4	0.048 (3)	0.033 (2)	0.033 (3)	0.0037 (19)	−0.002 (2)	−0.0031 (16)
C9	0.042 (3)	0.040 (2)	0.048 (3)	0.002 (2)	0.012 (2)	0.005 (2)
C8	0.058 (3)	0.041 (2)	0.036 (3)	0.000 (2)	0.010 (2)	−0.0029 (19)
C2	0.040 (3)	0.049 (3)	0.043 (3)	−0.001 (2)	0.013 (2)	−0.004 (2)

### Geometric parameters (Å, °)

**Table:**

Br1—C10	1.889 (5)	C1—H1B	0.9300
S1—C1	1.705 (5)	C3—C4	1.374 (7)
S1—C4	1.732 (5)	C3—C2	1.397 (6)
C6—N2	1.296 (6)	C3—H3A	0.9300
C6—C7	1.450 (7)	C11—C12	1.366 (7)
C6—H6A	0.9300	C11—H11A	0.9300
N2—N1	1.378 (5)	C7—C8	1.387 (6)
C10—C9	1.360 (6)	C7—C12	1.391 (6)
C10—C11	1.410 (7)	C12—H12A	0.9300
N1—C5	1.347 (6)	C9—C8	1.387 (7)
N1—H1A	0.8600	C9—H9A	0.9300
O1—C5	1.236 (5)	C8—H8A	0.9300
C5—C4	1.464 (6)	C2—H2B	0.9300
C1—C2	1.349 (7)		
			
C1—S1—C4	90.8 (2)	C12—C11—H11A	120.5
N2—C6—C7	120.1 (4)	C10—C11—H11A	120.5
N2—C6—H6A	120.0	C8—C7—C12	118.0 (4)
C7—C6—H6A	120.0	C8—C7—C6	119.6 (4)
C6—N2—N1	116.4 (4)	C12—C7—C6	122.4 (4)
C9—C10—C11	120.2 (5)	C11—C12—C7	121.8 (4)
C9—C10—Br1	120.7 (4)	C11—C12—H12A	119.1
C11—C10—Br1	119.1 (4)	C7—C12—H12A	119.1
C5—N1—N2	121.4 (4)	C3—C4—C5	121.7 (4)
C5—N1—H1A	119.3	C3—C4—S1	110.6 (3)
N2—N1—H1A	119.3	C5—C4—S1	127.7 (4)
O1—C5—N1	118.5 (4)	C10—C9—C8	119.9 (4)
O1—C5—C4	119.6 (4)	C10—C9—H9A	120.1
N1—C5—C4	121.9 (4)	C8—C9—H9A	120.1
C2—C1—S1	113.3 (4)	C7—C8—C9	121.1 (4)
C2—C1—H1B	123.4	C7—C8—H8A	119.4
S1—C1—H1B	123.4	C9—C8—H8A	119.4
C4—C3—C2	113.2 (4)	C1—C2—C3	112.1 (4)
C4—C3—H3A	123.4	C1—C2—H2B	124.0
C2—C3—H3A	123.4	C3—C2—H2B	124.0
C12—C11—C10	119.0 (4)		

### Hydrogen-bond geometry (Å, °)

**Table:**

*D*—H···*A*	*D*—H	H···*A*	*D*···*A*	*D*—H···*A*
N1—H1A···O1^i^	0.86	2.00	2.828 (5)	161

Symmetry codes: (i) −*x*−2, −*y*+1, −*z*.
